# Prognostic factors in refractory metastatic colorectal cancer patients treated with Trifluridine/Tipiracil

**DOI:** 10.1007/s00432-023-04909-6

**Published:** 2023-06-15

**Authors:** Agnieszka Koper, Sławomir Wileński, Paulina Śledzińska, Marek Bebyn, Krzysztof Koper

**Affiliations:** 1 Department of Oncology, Nicolaus Copernicus University in Torun, Ludwik Rydygier Collegium Medicum, 85-067 Bydgoszcz, Poland; 2 Department of Oncology, Franciszek Lukaszczyk Oncology Centre, 85-796 Bydgoszcz, Poland; 3 Department of Pharmaceutical Technology, Nicolaus Copernicus University in Torun, Ludwik Rydygier Collegium Medicum, 85-067 Bydgoszcz, Poland; 4 Central Cytostatic Drug Department, Hospital Pharmacy, The F. Lukaszczyk Oncology Centre, 85-796 Bydgoszcz, Poland; 5 10th Military Research Hospital and Polyclinic, 85-681 Bydgoszcz, Poland; 6 Department of Clinical Oncology, and Nursing, Department of Oncological Surgery, Nicolaus Copernicus University in Torun, Ludwik Rydygier Collegium Medicum, 85-067 Bydgoszcz, Poland

**Keywords:** Chemotherapy, Colorectal cancer, Prognostic factors, Trifluridine/Tipiracil

## Abstract

**Purpose:**

The systemic treatment options for metastatic colorectal cancer (mCRC) are unsatisfactory, and the disease recurs despite the use of numerous medications and their combinations. Trifluridine/Tipiracil is a relatively new drug used in refractory mCRC. Little is known about its real-world effectiveness and prognostic and predictive factors. Therefore, this study aimed to develop a prognostic model for refractory mCRC treated with Trifluridine/Tipiracil.

**Methods:**

We retrospectively evaluated the data from 163 patients who had received Trifluridine/Tipiracil as a third or fourth line of treatment for refractory mCRC.

**Results:**

After starting Trifluridine/Tipiracil, 21.5% of patients survived one year, and the median overall survival after Trifluridine/Tipiracil initiation was 251 days (SD: 17.855; 95%CI: 216–286). Median progression-free survival after Trifluridine/Tipiracil initiation was 56 days (SD: 4.826; 95%CI 47–65). Moreover, the median overall survival from diagnosis was 1333 days (SD: 82.84; 95%CI: 1170–1495). In forward stepwise multivariate Cox regression analysis, initial radical treatment (HR = 0.552, 95% CI 0.372–0.819, *p* < 0.003), the number of cycles of first-line chemotherapy (HR = 0.978, 95% CI 0.961–0.995, *p* < 0.011), the number of cycles of second-line chemotherapy (HR = 0.955, 95% CI 0.931–0.98, *p* < 0.011), *BRAF* mutation (HR = 3.016, 95% CI = 1.207–7.537, *p* = 0.018), and hypertension (HR = 0.64, 95% CI = 0.44–0.931, *p* = 0.02) were all associated with survival after Trifluridine/Tipiracil initiation. Our model and model-based nomogram displayed an AUC of 0.623 for one-year survival estimation in the testing cohort. The C-index for the prediction nomogram was 0.632.

**Conclusion:**

We have developed a prognostic model for refractory mCRC treated with Trifluridine/Tipiracil based on five variables. Moreover, we reported a nomogram which could be used by oncologists in clinic visits on a daily basis.

**Graphical abstract:**

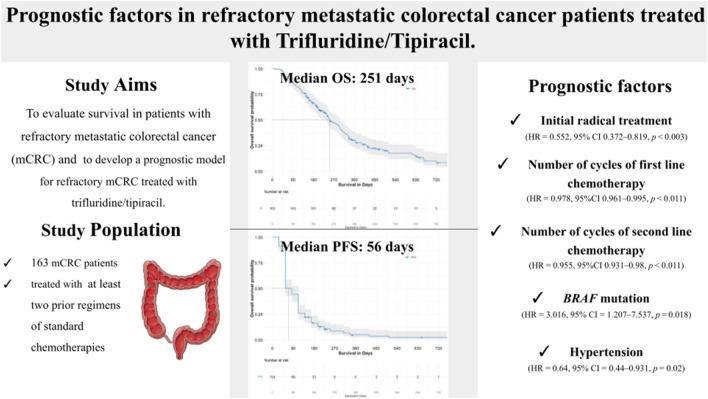

## Introduction

Colorectal cancer (CRC) is currently one of the most common malignancies worldwide (Siegel, Miller, and Jemal, 2020b). The death rate from colorectal cancer is decreasing and is now approximately 50% of its highest levels (Siegel et al. 2020a; b). This trend is attributed to the increased utilization of screening tests (fecal occult blood test and colonoscopy) and the improvement of treatment options for these patients (Siegel et al. 2020a). The fundamental systemic treatment for colorectal cancer is fluoropyrimidines (Meyerhardt and Mayer 2005). The systemic therapy of colorectal cancer is based on the combination of fluorouracil with oxaliplatin or irinotecan. When the tumor has the wild-type *RAS* gene, an epidermal growth factor inhibitor (e.g., cetuximab or panitumumab) might improve treatment for metastatic colorectal cancer (Mayer et al. 2015). On the other hand, in *RAS*-mutant disease, bevacizumab-based chemotherapy is considered the best choice (Cervantes et al. 2023). Systemic therapeutic options for metastatic colorectal cancer are currently insufficient. Patients with metastatic colorectal cancer (mCRC) have a median overall survival (OS) of about 30 months, which is unsatisfactory and demands the search for new active therapies (Heinemann et al. 2014; Loupakis et al. 2014; Yamada et al. 2013). For many of these individuals, the hunt for new and effective medications is still underway.

The results of the pivotal RECOURSE study showed that Trifluridine/Tipiracil, compared to placebo, prolongs survival and reduces the risk of death in patients with refractory mCRC. The drug, previously known as TAS-102, was administered to adult patients with mCRC who had already received or were ineligible for various treatments, including fluoropyrimidine, oxaliplatin, and irinotecan chemotherapy, and therapy with VEGF inhibitors and EGFR inhibitors (Fukushima et al. 2000). Oral Trifluridine/Tipiracil consists of the thymidine-based nucleic acid analog trifluridine and the thymidine phosphorylase inhibitor tipiracil hydrochloride. The drug's dual mode of action enhances its therapeutic efficacy (Mayer et al. 2015). As a result of the publication of promising study results, a marketing authorization for Trifluridine/Tipiracil was granted in 2019 (Fukushima et al. 2000).

There are prognostic markers for metastatic colorectal cancer in the literature, such as fewer metastases and no extrahepatic metastases (EORTC 40983). However, there are limited data on the OS of Trifluridine/Tipiracil-treated patients and the factors that contribute to their longer survival are still unknown. Therefore, we retrospectively evaluated the efficacy and safety of Trifluridine/Tipiracil in patients with refractory mCRC. The purpose of this study was to identify prognostic markers for Trifluridine/Tipiracil-treated individuals.

## Materials and methods

### Patients

We retrospectively evaluated the data from 163 patients who had received Trifluridine/Tipiracil as a third or fourth line of treatment for mCRC at the Oncology Center in Bydgoszcz, Poland from November 2019 to May 2022.

Inclusion criteria were (1) histologically confirmed unresectable colorectal adenocarcinoma; (2) refractory disease or intolerant to fluoropyrimidines, oxaliplatin, irinotecan, anti-VEGF antibodies, and anti-EGFR antibodies (if *KRAS* exon 2 wild-type tumor); (3) no previous treatment with Trifluridine/Tipiracil; (4) Eastern Cooperative Oncology Group Performance Status (ECOG-PS) 0 or 1; and (5) adequate bone marrow, hepatic, and renal function; (6) as a third or fourth line of chemotherapy; and (7) patients older than 18 years. The study protocol was approved by the Ethics Committee at Collegium Medicum in Bydgoszcz, Nicolaus Copernicus University in Torun (approval number: KB 16/2023).

### Treatment

The starting dose of Trifluridine/Tipiracil (35 mg/m2) was administered twice daily on Days 1–5 and Days 8–12 of each 28-day cycle as long as benefit is observed or until unacceptable toxicity occurs (Doi et al. 2012). Both regimens were repeated every 4 weeks. The treatments were continued until disease progression, death, unacceptable toxicity, or patient refusal. We included patients whose initial dose had been reduced by physician decision in the present study. Radical intention in our cohort means that patients were suitable to receive operation with or without preoperative chemoradiotherapy, and tumors were potentially respectable initially. Progression-free survival (PFS) was assessed after each two cycles using medical history, imaging and laboratory tests.

### Data collection

Clinical pathological characteristics and treatment data were collected from eligible patients’ medical records, including survival status, sex, age, comorbidities, and ECOG-PS. Disease characteristics included *RAS* and *BRAF* mutational status, primary tumor location, histological grade, presence of liver or lung metastasis, the interval from disease diagnosis to Trifluridine/Tipiracil initiation as well as treatment history, including primary tumor resection, and previous treatments received, including type of first, second and/or third line of chemotherapy, number of cycles of each line of chemotherapy. Lastly, adverse events due to Trifluridine/Tipiracil administration were also collected from patient records for analysis.

### Statistical analysis

Statistical analyses were performed using R and Google Sheets. Means and standard deviation (SD) were used to describe normally, and non-normally distributed data. Overall survival after Trifluridine/Tipiracil was calculated from the date of initiation of Trifluridine/Tipiracil to the date of death. The OS curves were plotted using the Kaplan–Meier technique. The findings were measured using 95% confidence intervals (95% CI), and a *p* value of 0.05 was considered statistically significant. A model was created to determine advantageous prognostic factors for longer survival of refractory mCRC patients using stepwise forward multivariate Cox regression. Factors that were found to be associated with prolonged survival were determined from the data, based on the criteria of being both clinically relevant and easily obtainable at the start of Trifluridine/Tipiracil treatment.

## Results

Participants in the study included 163 patients who were diagnosed with refractory mCRC and received treatment with Trifluridine/Tipiracil at the Franciszek Łukaszczyk Oncology Centre in Bydgoszcz, Poland, between the years 2019 and 2022. Of the 163 diagnosed mCRC patients, ninety-eight (60.1%) were men and sixty-five (39.9%) were women. The median age at the onset of the disease was 64 (SD: 10) years. The most common locations for mCRC were: colon (51.5%), recto-sigmoid flexure (10.4%), and rectum (38%). The majority of tumors were left-sided (79.8%). Most patients had metastases in the liver, and approximately half of the patients had metastases in the lungs. The *KRAS* mutation occurred in 60.5% of patients. However, only 6.2% and 3.1% of patients had mutations in *NRAS* and *BRAF*, respectively. Diabetes mellitus type 2 (DMT2) and hypertension occurred in 12.4% and 41.7% of patients, respectively. Ten patients are still being treated at the time of data collection. Two patients got more than 20 cycles of Trifluridine/Tipiracil (Table 1).

**Table 1:** Patients characteristics

	*N*	%
Treatment status		
Completed	152	93.3
Underway	11	6.7
Living patients: number of cycles		
4	2	18.2
5	3	27.3
6	1	9.1
7	1	9.1
8	1	9.1
10	1	9.1
21	1	9.1
28	1	9.1
Gender		
Woman	65	39.9
Man	98	60.1
ICD10		
C.18	84	51.5
C.19	17	10.4
C.20	62	38.0
DMT2		
No	141	87.6
Yes	20	12.4
Hypertension		
No	95	58.3
Yes	68	41.7
BMI (mean; SD)		
	27	4,6
Liver metastasis		
No	27	16.6
Yes	136	83.4
Lung metastasis		
No	90	55.2
Yes	73	44.8
Other metastasis		
No	124	76.1
Yes	39	23.9
ECOG-PS		
1	163	100.0
KRAS mutation		
No	64	39.5
Yes	98	60.5
NRAS mutation		
No	152	93.8
Yes	10	6.2
BRAF mutation		
No	158	96.9
Yes	5	3.1
Grade		
G1	7	4.3
G2	152	93.3
G3	4.0	2.5
Side		
Left	130	79.8
Right	33	20.2
Age (mean; SD)		
	64.0	10.0

Eastern Cooperative Oncology Group Performance Status (ECOG-PS) Diabetes mellitus type 2 (DMT2)

Over a third of patients were initially treated with radical intent. Moreover, 12.3% of patients had neoadjuvant treatment, and 29.4% had adjuvant therapy. As a first line of treatment, about 70% of patients received FOLFIRI-based chemotherapy, and almost half received only FOLFIRI. As a second line of treatment, 63.8% of patients received FOLFOX-based chemotherapy. Lastly, 16.6% of patients also received a third line of chemotherapy prior to Trifluridine/Tipiracil, most of which was panitumumab (Table 2).

**Table 2:** Treatment prior to Trifluridine/Tipiracil

	*N*	%
Radical treatment		
No	103	63.2
Yes	60	36.8
Neoadjuvant treatment		
No	143	87.7
Yes	20	12.3
Type of neoadjuvant treatment		
None	143	87.7
RT + CHT	14	8.6
RTH	5	3.1
CHT	1	0.6
Adjuvant treatment		
No	115	70.6
Yes	48	29,40
Type of adjuvant treatment		
None	115	70.6
LF4	25	15.3
FOLFOX-4	17	10.4
XELOX	4	2.5
CAPECITABINE	2	1.2
First-line treatment of metastatic disease		
FOLFIRI	81	49.7
FOLFOX-4	32	19.6
FOLFIRI + CETUXIMAB	28	17.2
XELOX	8	4.9
FOLFOX-4 + PANITUMUMAB	7	4.3
FOLFIRI + BEVACIZUMAB	6	3.7
FOLFOX-4 + BEVACIZUMAB	1	0.6
Termination cause of first-line treatment		
Complications	5	3.1
Progression	158	96.9
Number of cycles of first-line chemotherapy (Mean; SD)	15.4	12.6
Type of second-line treatment		
FOLFOX-4	61	37.4
FOLFOX-4 + BEVACIZUMAB	43	26.4
FOLFIRI	39	23.9
FOLFIRI + AFLIBERCEPT	9	5.5
XELOX	6	3.7
LF4	4	2.4
IRINOTEKAN	1	0.6
Termination cause of second-line treatment		
Complications	3	1.8
Progression	160	98.2
Number of cycles of second-line chemotherapy (Mean; SD)	11.6	7.2
Type of third-line treatment		
None	136	83.4
PANITUMUMAB	10	6.1
XELOX	10	6.1
FOLFIRI	4	2.5
FOLFOX-4	2	1.2
CETUXIMAB	1	0.6
Termination cause of third-line treatment		
None	136	83.4
Progression	27	16.6
Number of cycles of third line chemotherapy (Mean; SD)	11.6	7.2

*CHT* chemotherapy, *RTH* Radiotherapy

**Fig. 1:**
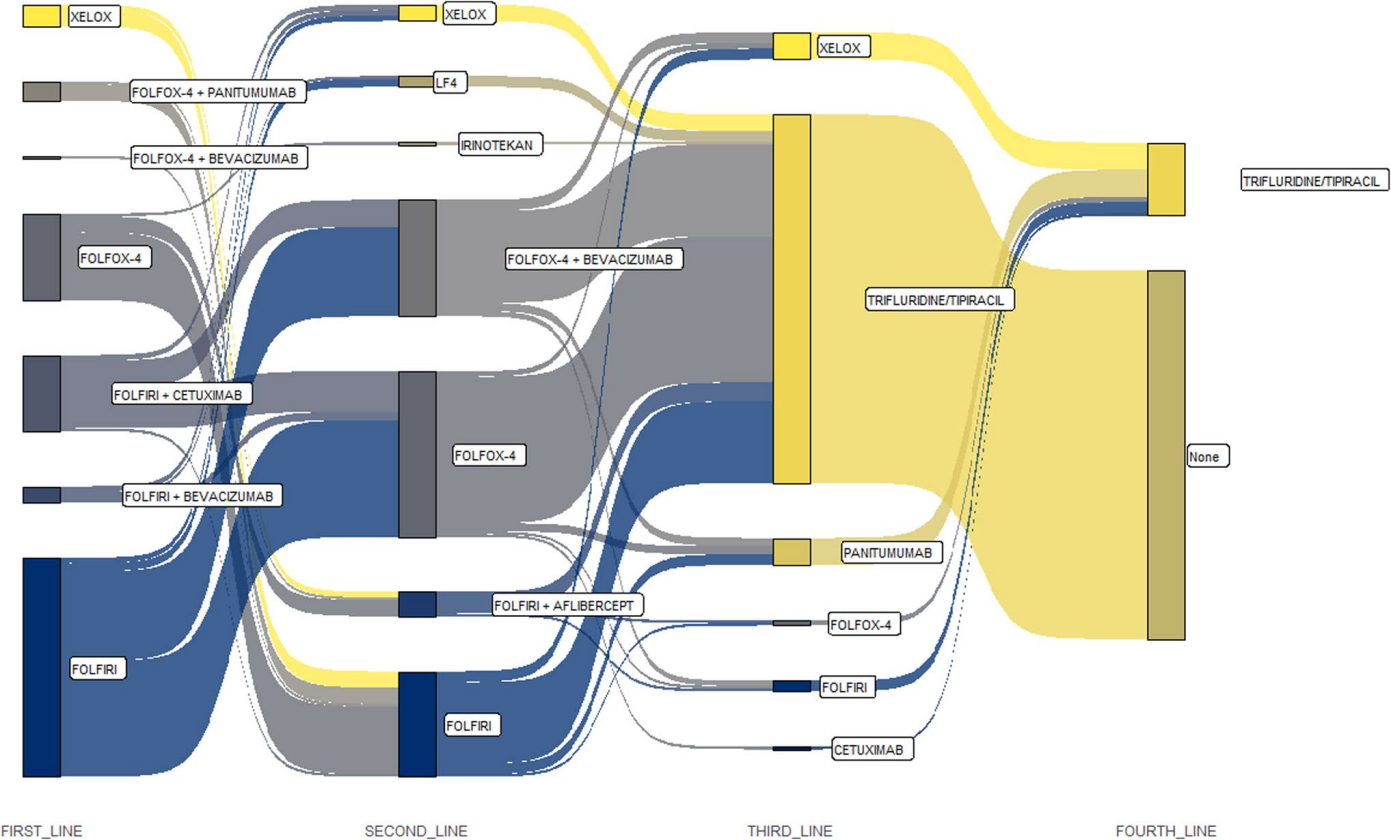
Sankey chart of patients’ treatment

Almost all patients who received FOLFIRI in the first line of treatment received FOLFOX-based chemotherapy in the second line. On the other hand, most patients who received FOLFOX or XELOX in the first line of chemotherapy received FOLFIRI-based chemotherapy in the second line. The course of treatment is presented in Fig. 1.

**Fig. 2:**
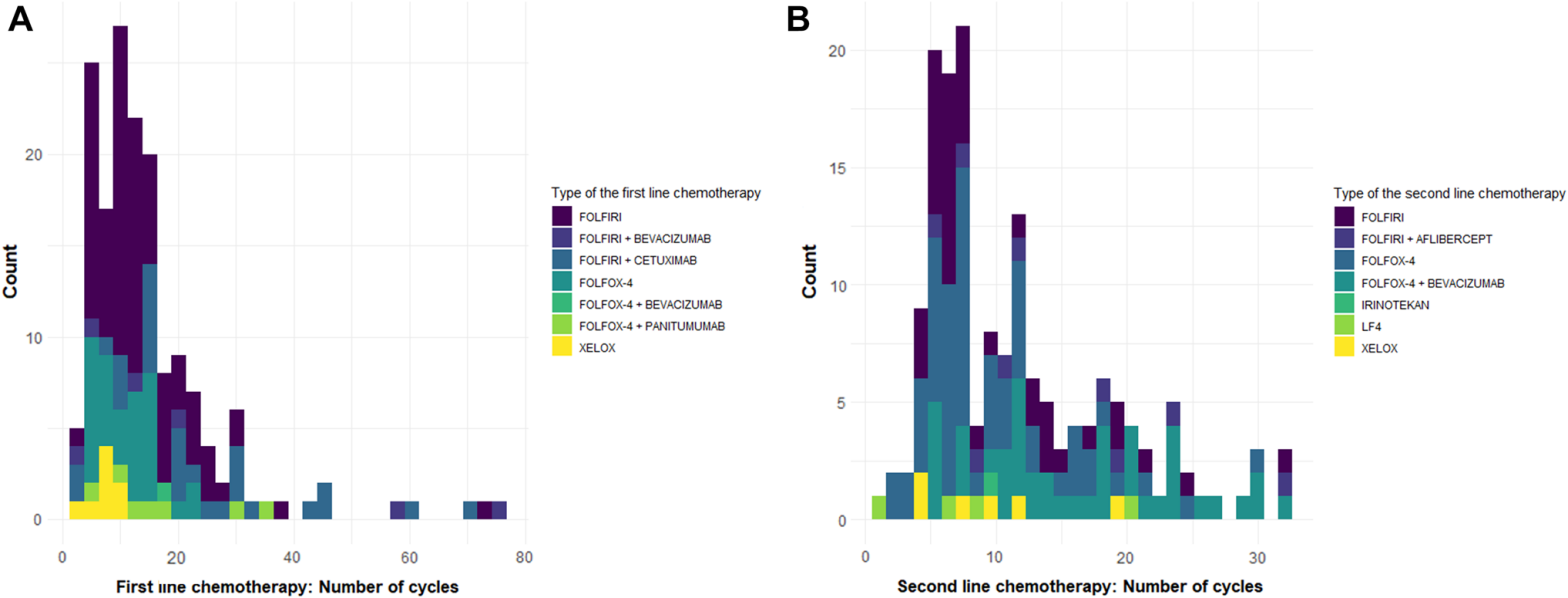
Histogram of the first-line (**A**) and second-line (**B**) chemotherapy cycles concerning the type of chemotherapy

There is a pattern that patients who received FOLFIRI-based chemotherapy in the first line of chemotherapy were treated longer with the first line compared to FOLFOX-based chemotherapy (Fig. 2A). In second-line treatment, there is no tendency for any one type of chemotherapy to be associated with a more extended number of cycles. (Fig. 2B).

About half of the patients received more than two doses of Trifluridine/Tipiracil. The main reason for discontinuing treatment with Trifluridine/Tipiracil was a progression, which occurred in 75.5% of patients. During therapy with Trifluridine/Tipiracil, anemia occurred in 75% of cases. Moreover, leukopenia and neutropenia occurred in approximately 50% of patients (Table 3).

**Table 3:** Trifluridine/Tipiracil treatment

	*N*	%
Trifluridine/Tipiracil—number of cycles		
2	64	39.3
4	29	17.8
1	19	11.7
3	10	6.1
Still in treatment	10	6.1
6	8	4.9
5	6	3.7
7	5	3.1
8	4	2.5
9	2	1.2
11	2	1.2
12	2	1.2
15	1	0.6
18	1	0.6
Termination cause of Trifluridine/Tipiracil treatment		
Progression	123	75.5
Still receiving	10	
Death	2	
NA	19	17.8
Complications	9	6.7
The first dose reduction occurred in the cycle:		
Without reduction	136	83.4
2	12	7.4
3	8	4.9
4	2	1.2
5	2	1.2
6	2	1.2
10	1	0.6
The second dose reduction occurred in the cycle:		
Without reduction	157	96.3
4	2	1.2
5	2	1.2
3	1	0.6
6	1	0.6
Leucopenia		
None	73	46.5
G1	30	19.1
G2	41	26.1
G3	13	8.3
Neutropenia		
None	76	48.4
G1	10	6.4
G2	31	19.7
G3	34	21.7
G4	6	3.8
Anemia		
None	38	24.1
G1	68	43.0
G2	41	25.9
G3	11	7.0
Thrombocytopenia		
None	136	86.1
G1	17	10.8
G2	5	3.2
Fatigue		
No	126	77.3
Yes	37	22.7
Other complications		
	32	19.5

*NA* not available

**Fig. 3:**
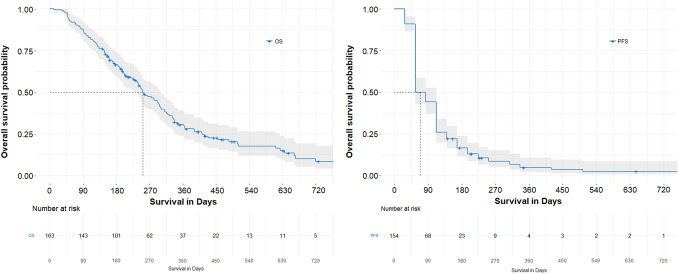
**A** Kaplan–Meier curve of overall survival (OS) after Trifluridine/Tipiracil initiation. **B** Kaplan–Meier curve of progression-free survival (PFS) after Trifluridine/Tipiracil initiation

After starting Trifluridine/Tipiracil, 21.5% of patients survived one year and median overall survival after Trifluridine/Tipiracil initiation was 251 days (SD: 17.855; 95%CI: 216–286) (Fig. 3A). The median PFS after Trifluridine/Tipiracil initiation was 56 days (SD: 4.826; 95%CI 47–65) (Fig. 3B). Moreover, the median OS from diagnosis was 1333 days (SD: 82.84; 95%CI: 1170–1495).

In forward stepwise multivariate Cox regression analysis, initial radical treatment (HR = 0.552, 95% CI 0.372–0.819, *p* < 0.003), the number of cycles of first-line chemotherapy (HR = 0.978, 95% CI 0.961–0.995, *p* < 0.011), the number of cycles of second-line chemotherapy (HR = 0.955, 95% CI 0.931–0.98, *p* < 0.011), *BRAF* mutation (HR = 3.016, 95% CI = 1.207–7.537, *p* = 0.018), and hypertension (HR = 0.64, 95% CI = 0.44–0.931, *p* = 0.02) were all associated with survival after Trifluridine/Tipiracil initiation (Table 4).

**Table 4:** Forward stepwise multivariable Cox regression predicting survival after Trifluridine/Tipiracil initiation

	B	SE	Wald	df	Sig	Exp(B)	95% CI for Exp(B)	
							Lower	Upper
*BRAF* mutation	1.104	0.467	5.583	1	0.018	3.016	1.207	7.537
Radical treatment initially	− 0.594	0.202	8.695	1	0.003	0.552	0.372	0.819
Number of cycles of first-line chemotherapy	− 0.022	0.009	6.475	1	0.011	0.978	0.961	0.995
Number of cycles of second-line chemotherapy	− 0.046	0.013	12.395	1	0	0.955	0.931	0.98
Hypertension	− 0.447	0.191	5.454	1	0.02	0.64	0.44	0.931

*BRAF* mutation, radical treatment initially, the number of cycles of first- and second-line treatment and hypertension were incorporated into the survival prediction model. The model predictors with coefficients are presented in Table 4. On the basis of the model, a nomogram was constructed.

Nomograms are valuable because they transfer predicted probabilities onto a scale from 0 to 100 using an intuitive graphical interface. The total number of points accumulated by the various factors corresponds to a patient's predicted likelihood (Diblasio and Kattan 2003). The point system works by ranking the effect estimates, regardless of statistical significance, and the presence of other covariates influences it.

**Fig. 4:**
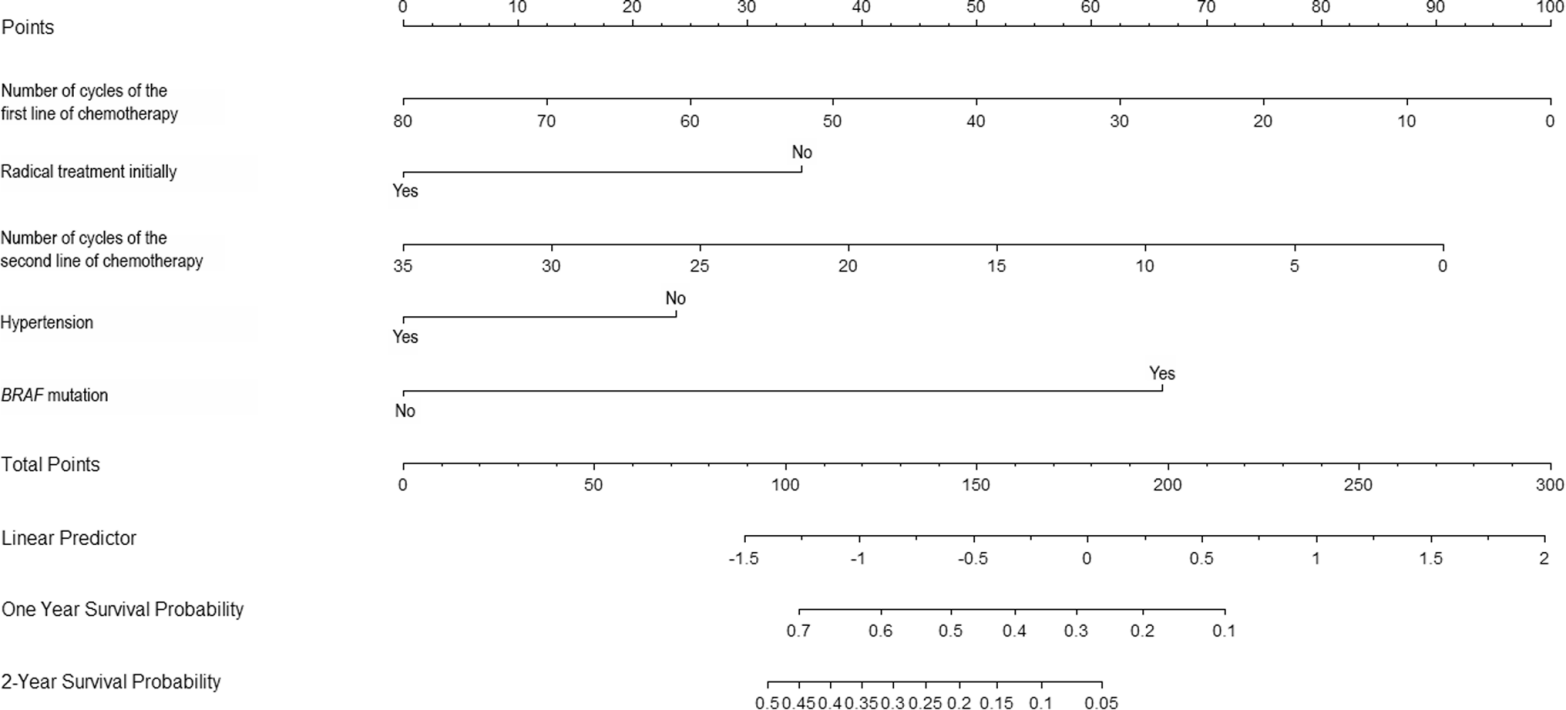
Nomogram for 1- and 2-year survival among patients with colorectal cancer treated with Trifluridine/Tipiracil. Instructions: locate the number of cycles of first-line treatment on the initial age axis. Draw a line straight upward to the point’s axis to determine how many points the patient receives. Repeat the process for each variable. Sum the points achieved for each of the predictors. Locate the final sum on the total points axis. Draw a straight line to find the patient’s probability of 1- and 2-year survival

Figure 4 shows our nomogram consisting of five carefully chosen variables. In our model, the number of cycles of first-line chemotherapy has the highest effect and, thus, is correlated with the highest number of points. In the case of radical treatment initially, number of cycles of second-line chemotherapy, hypertension and *BRAF* mutation, a patient with 40 cycles of first-line chemotherapy, without radical treatment initially, with 35 cycles of second-line chemotherapy, no mutation in *BRAF* gene, and without hypertension is assigned 50, 35, 0, 25 and 0 points, respectively, which gives 110 points in total which corresponds to 0.68 and 0.4 of one- and two-year survival probability after Trifluridine/Tipiracil initiation. Regardless of statistical significance, the variable with the highest effect will be assigned 100 points on the scale. The remaining variables are assigned fewer points proportional to their effect size.

Nomograms rank the importance of an impact in predicting the outcome only in the context of the other covariates currently in the model. The number of points does not reflect the association with the outcome in a broader sense, nor does it represent statistical significance in *p* values (Iasonos et al. 2008).

### Model performance

**Fig. 5:**
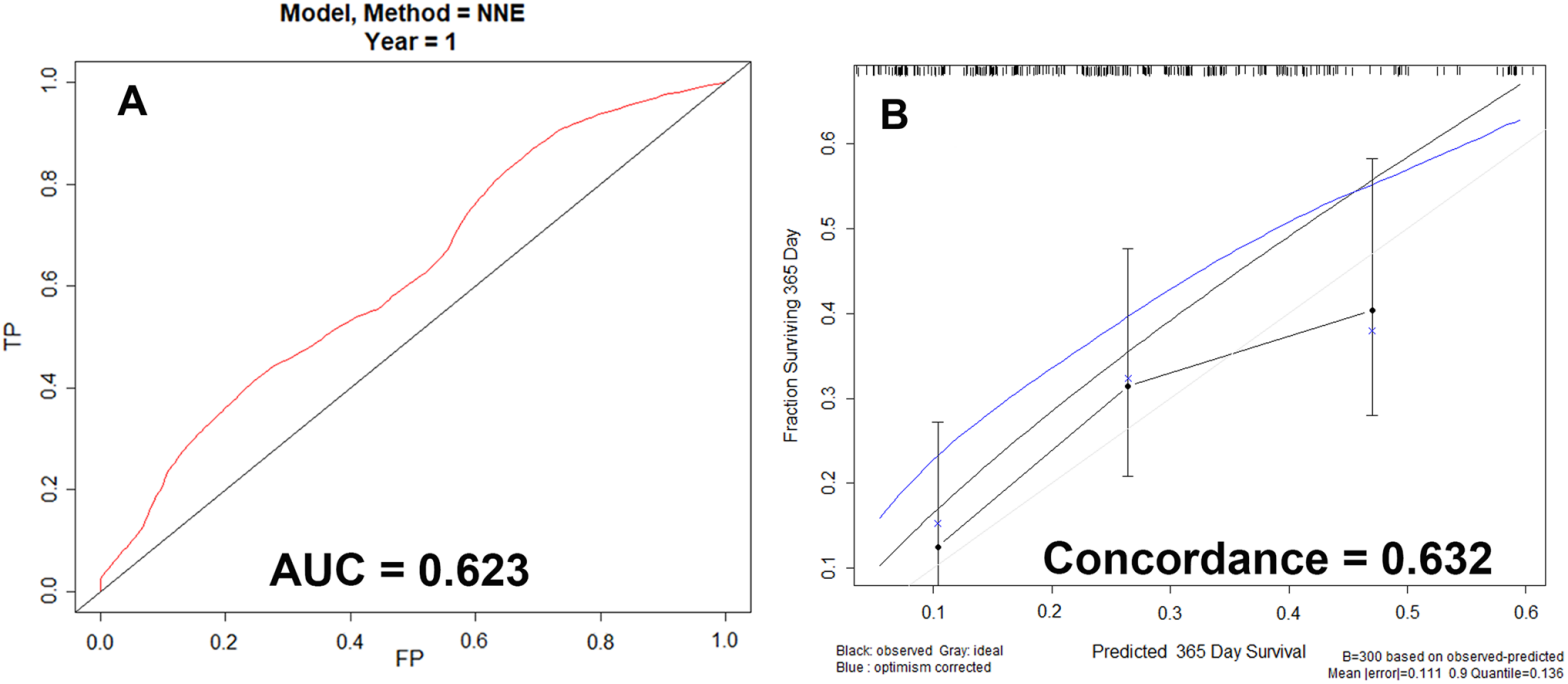
Performance of the model predicting survival after Trifluridine/Tipiracil initiation. **A** Receiver operating characteristic (ROC) curves for the model **B** Calibration plot of the model. Calibration curves depict the calibration of the model in terms of the agreement between the predicted risks of death and the observed deaths. The y-axis represents the actual one-year survival rate. The x-axis represents the predicted one-year survival probability. The dashed line (the 45-degree diagonal line) represents a perfect prediction by an ideal model. The solid black line represents the performance of the nomogram, in which a closer fit to the diagonal dotted line represents a better prediction

Our model and model-based nomogram model displayed an AUC of 0.623 for one-year survival estimation in the testing cohort. The C-index for the prediction nomogram was 0.632. The calibration plot showed that the predicted risks of death of the nomogram were in good accordance with the actual risks for death (Fig. 5).

## Discussion

The use of Trifluridine/Tipiracil as a treatment option for metastatic colorectal cancer is becoming more common. However, there is limited information available on the survival outcomes of patients after starting Trifluridine/Tipiracil treatment, and on the factors that impact patient survival. In this study, we provide the results of an analysis of a large group of refractory mCRC patients, one of the largest groups described to date.

We found that 21.5% of patients survived one year after beginning treatment with Trifluridine/Tipiracil, with a median overall survival of 8.5 months and PFS of 1.9 months. The median OS in our cohort was comparable to that from the RECOURSE trial (8.5 months vs 7.1 months) or TERRA trail (7.8 months), or other reported studies (6.5–9.0 months) (Carriles et al. 2019; Masuishi et al. 2017; Mayer et al. 2015; Xu et al. 2018; Yoshino et al. 2012).

In our study, we found that the variables that had the strongest association with survival were initial radical treatment, the number of cycles of first- and second-line chemotherapy, *BRAF* mutation status, and the presence of hypertension. Some of these factors were also found to be associated with survival in other models of refractory mCRC. For example, Tabernero et al. found that factors including neutrophil–lymphocyte ratio (NLR), CEA, ECOG-PS, number of metastatic sites, time since diagnosis of metastasis until starting Trifluridine/Tipiracil, and alkaline phosphatase were associated with survival in their model (Tabernero et al. 2020). The Colon Life nomogram used four factors in its model, including ECOG-PS, primary tumor resection, LDH value, and peritoneal involvement (Pietrantonio et al. 2017). Additionally, the TAS-RECOSMO model included six variables, such as ECOG-PS, KRAS/NRAS/BRAF mutation status, the time between diagnosis of metastasis and the start of Trifluridine/Tipiracil treatment, NLR, CEA, and alkaline phosphatase (Fernández Montes et al. 2021).

In presented study, initial radical treatment was a favorable prognostic factor (HR = 0.552, 95% CI 0.372–0.819, *p* < 0.003). Radical intention in our cohort means that patients were suitable to receive operation with or without preoperative chemoradiotherapy and tumors were potentially respectable initially. Our findings align with those of the RAXO study, a nationwide prospective study, which found that patients with resectable cancer had median survival rates that were two to three times higher than those who were treated with systemic therapy alone (Osterlund et al. 2021).

Our study found that patients who received a greater number of cycles of first-line chemotherapy had a better prognosis (HR = 0.978, 95% CI 0.961–0.995, *p* < 0.011). Similarly, patients who received a greater number of cycles of second-line chemotherapy had a longer survival time (HR = 0.955, 95% CI 0.931–0.98, *p* < 0.011). This corresponds to a longer time from diagnosis to starting TAS-102 treatment, which has been found to be a positive predictor of survival (Fernández Montes et al. 2021; Moriwaki et al. 2020). The variable of time from diagnosis to initiation of Trifluridine/Tipiracil therapy appears to have a slight non-linear relationship, with initial protection that is quickly negated by increased risk for therapies administered at later stages and in more advanced forms of cancer (Fernández Montes et al. 2021).

A mutation in the *BRAF* gene was associated with a worse prognosis in our population (HR = 3.016, 95% CI = 1.207–7.537, *p* = 0.018). The presence of a *BRAF* mutation has previously been identified as a negative prognostic indicator and a predictor of poor response to treatment with EGFR blockade drugs such as panitumumab or cetuximab, as well as to BRAF inhibitors like vemurafenib (Cervantes et al. 2023; Di Nicolantonio et al. 2008; Prahallad et al. 2012; Tol et al. 2009). In CRC, there is a high synergy between *EGFR* blockage and *BRAF*(V600E) inhibition. Hence, blocking EGFR inhibits *BRAF*(V600E), resulting in cell proliferation. *BRAF*(V600E) inhibition, on the other hand, induces a rapid feedback activation of EGFR, which enables sustained proliferation in the presence of *BRAF*(V600E) inhibition (Prahallad et al. 2012). To effectively treat BRAF-mutated mCRC, a combination of *EGFR* blocking and *BRAF* inhibition (such as encorafenib–cetuximab) is required. However, none of our cohort's patients received encorafenib–cetuximab.

In our model, hypertension was a favorable prognostic factor (HR = 0.64, 95% CI = 0.44–0.931, *p* = 0.02). Hypertension is a typical adverse effect of anti-VEGF antibody therapy (such as bevacizumab) and a hallmark of VEGF signaling pathway suppression (Ranpura et al. 2010). Österlund et al. reported that hypertension was linked with increased progression-free survival (PFS) (10.5 vs. 5.3 months; *p* = 0.008) and OS (25.8 vs. 11.7 months; *p* = 0.001) in mCRC patients treated with bevacizumab-containing chemotherapy (Österlund et al. 2011). Scartozzi et al. also observed that hypertension was related to considerably prolonged OS and PFS in patients with mCRC treated with bevacizumab (Scartozzi et al. 2009). Nonetheless, we are the first to report that hypertension is also a favorable prognostic factor for Trifluridine/Tipiracil therapy.

In the testing cohort, our model and model-based nomogram model had an AUC of 0.623 and a c-index of 0.632 for estimating one-year survival. In addition, the calibration plot demonstrated that the predicted death risks of the nomogram were in good agreement with the actual mortality rate. Our result is equivalent to those previously published in the scientific literature. The bootstrapped bias-corrected c-index for the TAS-RECOSMO model was 0.682. The c-index values for the Colon Life model, FAS-CORRECT, and RECOURSE were 0.69, 0.63, and 0.507, respectively (Fernández Montes et al. 2021).

In our study group, 25.5% of patients had neutropenia of grade 3 or above, whereas 8.3% had leukopenia of the same severity. In addition, 7% of the population had anemia of grade 3 or above. In the RECOURSE study, 38% of patients experienced neutropenia of grade 3 or above. The reported side events were less severe than in the RECOURSE trial. Twenty-one percent of patients had leukopenia of grade 3 or higher. The incidence of anemia of grade 3 or higher was 18% (Mayer et al. 2015). Interestingly, chemotherapy-induced neutropenia (CIN) may be a helpful predictor of treatment outcomes for Trifluridine/Tipiracil-treated individuals. In RECOURSE, trifluridine exposure was associated with an elevated CIN risk. Patients treated with Trifluridine/Tipiracil who had CIN had improved OS and PFS compared to those in the placebo group and those who did not develop CIN. The J003 cohort reported comparable results, supporting the RECOURSE findings (Yoshino et al. 2020). In addition, a publication from a single institution demonstrated a tendency toward improved OS for patients who experienced chemotherapy-induced neutropenia at 1 month (CIN-1-month) (Kasi et al. 2016). However, we did not include CIN in our predictive model because this metric is unavailable at the beginning of Trifluridine/Tipiracil therapy.

### Limitations of the study

The present study had several limitations. First, it was a retrospective nonrandomized analysis. In addition, we only included patients with an ECOG 0 or 1 because this was required to receive government reimbursement for Trifluridine/Tipiracil treatment. Furthermore, we were unable to include data on mismatch repair and microsatellite instability status in our analysis. In addition, we have no data on the evaluation of dihydropyrimidine dehydrogenase activity. Despite these limitations, the study provides valuable information on the use of Trifluridine/Tipiracil in the treatment of metastatic colorectal cancer and the prognostic factors associated with patient survival.

## Conclusions

In conclusion, we have established a prognostic model for refractory mCRC treated with Trifluridine/Tipiracil based on five variables: initial radical treatment, the number of cycles of first- and second-line chemotherapy, *BRAF* gene mutation status, and presence of hypertension. These factors are accessible before the beginning of Trifluridine/Tipiracil. In addition, we reported a model-based nomogram that oncologists might employ during daily clinic visits. This real-world trial demonstrates the efficacy and safety of Trifluridine/Tipiracil in patients with refractory mCRC in routine clinical practice, with survival and tolerability outcomes broadly consistent with prior clinical and real-world studies in this setting.

## Data Availability

The datasets generated during and/or analyzed during the current study are available from the corresponding author on reasonable request.

## References

[CR1] Carriles C, Jimenez-Fonseca P, Sánchez-Cánovas M, Pimentel P, Carmona-Bayonas A, García T, Carbajales-Álvarez M, Lozano-Blázquez A (2019) Trifluridine/Tipiracil (TAS-102) for refractory metastatic colorectal cancer in clinical practice: a feasible alternative for patients with good performance status. Clin Trans Oncol 21(12):1781–1785. 10.1007/s12094-019-02154-3 10.1007/s12094-019-02154-3 31209792

[CR2] Cervantes A, Adam R, Roselló S, Arnold D, Normanno N, Taïeb J, Seligmann J, De Baere T, Osterlund P, Yoshino T, Martinelli E, ESMO Guidelines Committee (2023) Metastatic colorectal cancer: ESMO clinical practice guideline for diagnosis, treatment and follow-up. Ann Oncol 34(1):10–32. 10.1016/j.annonc.2022.10.003. (**clinicalguidelines@esmo.org**) 36307056 10.1016/j.annonc.2022.10.003

[CR3] Di Nicolantonio F, Martini M, Molinari F, Sartore-Bianchi A, Arena S, Saletti P, De Dosso S, Mazzucchelli L, Frattini M, Siena S, Bardelli A (2008) Wild-type BRAF is required for response to panitumumab or cetuximab in metastatic colorectal cancer. J Clin Oncol 26(35):5705–5712. 10.1200/JCO.2008.18.0786 19001320 10.1200/JCO.2008.18.0786

[CR4] Diblasio CJ, Kattan MW (2003) Use of nomograms to predict the risk of disease recurrence after definitive local therapy for prostate cancer. Urology 62(Suppl 1):9–18. 10.1016/j.urology.2003.09.029 14747038 10.1016/j.urology.2003.09.029

[CR5] Doi T, Ohtsu A, Yoshino T, Boku N, Onozawa Y, Fukutomi A, Hironaka S, Koizumi W, Sasaki T (2012) Phase I study of TAS-102 treatment in Japanese patients with advanced solid tumours. Br J Cancer 107(3):429–434. 10.1038/bjc.2012.274 22735906 10.1038/bjc.2012.274 PMC3405214

[CR6] Fernández Montes A, Carmona-Bayonas A, Jimenez-Fonseca P, Vázquez Rivera F, Martinez Lago N, Covela Rúa M, Cousillas Castiñeiras A, Gonzalez Villarroel P, De la Cámara Gómez J, Méndez JCM, Carriles Fernández C, Sanchez Cánovas M, Garcia García T (2021) Prediction of survival in patients with advanced, refractory colorectal cancer in treatment with trifluridine/tipiracil: real-world vs clinical trial data. Sci Rep. 10.1038/s41598-021-93732-5 34253805 10.1038/s41598-021-93732-5 PMC8275736

[CR7] Fukushima M, Suzuki N, Emura T, Yano S, Kazuno H, Tada Y, Yamada Y, Asao T (2000) Structure and activity of specific inhibitors of thymidine phosphorylase to potentiate the function of antitumor 2’-deoxyribonucleosides. Biochem Pharmacol 59(10):1227–1236. 10.1016/s0006-2952(00)00253-7 10736423 10.1016/s0006-2952(00)00253-7

[CR8] Heinemann V, von Weikersthal LF, Decker T, Kiani A, Vehling-Kaiser U, Al-Batran S-E, Heintges T, Lerchenmüller C, Kahl C, Seipelt G, Kullmann F, Stauch M, Scheithauer W, Hielscher J, Scholz M, Müller S, Link H, Niederle N, Rost A, Stintzing S (2014) FOLFIRI plus cetuximab versus FOLFIRI plus bevacizumab as first-line treatment for patients with metastatic colorectal cancer (FIRE-3): a randomised, open-label, phase 3 trial. Lancet Oncol 15(10):1065–1075. 10.1016/S1470-2045(14)70330-4 25088940 10.1016/S1470-2045(14)70330-4

[CR9] Iasonos A, Schrag D, Raj GV, Panageas KS (2008) How to build and interpret a nomogram for cancer prognosis. J Clin Oncol 26(8):1364–1370. 10.1200/JCO.2007.12.9791 18323559 10.1200/JCO.2007.12.9791

[CR10] Kasi PM, Kotani D, Cecchini M, Shitara K, Ohtsu A, Ramanathan RK, Hochster HS, Grothey A, Yoshino T (2016) Chemotherapy induced neutropenia at 1-month mark is a predictor of overall survival in patients receiving TAS-102 for refractory metastatic colorectal cancer: a cohort study. BMC Cancer 16:467. 10.1186/s12885-016-2491-y 27412464 10.1186/s12885-016-2491-y PMC4944251

[CR11] Loupakis F, Cremolini C, Masi G, Lonardi S, Zagonel V, Salvatore L, Cortesi E, Tomasello G, Ronzoni M, Spadi R, Zaniboni A, Tonini G, Buonadonna A, Amoroso D, Chiara S, Carlomagno C, Boni C, Allegrini G, Boni L, Falcone A (2014) Initial therapy with FOLFOXIRI and bevacizumab for metastatic colorectal cancer. N Engl J Med 371(17):1609–1618. 10.1056/NEJMoa1403108 25337750 10.1056/NEJMoa1403108

[CR12] Masuishi T, Taniguchi H, Hamauchi S, Komori A, Kito Y, Narita Y, Tsushima T, Ishihara M, Todaka A, Tanaka T, Yokota T, Kadowaki S, Machida N, Ura T, Fukutomi A, Ando M, Onozawa Y, Tajika M, Yasui H, Yamazaki K (2017) Regorafenib versus Trifluridine/Tipiracil for refractory metastatic colorectal cancer: a retrospective comparison. Clin Colorectal Cancer 16(2):e15–e22. 10.1016/j.clcc.2016.07.019 27670892 10.1016/j.clcc.2016.07.019

[CR13] Mayer RJ, Van Cutsem E, Falcone A, Yoshino T, Garcia-Carbonero R, Mizunuma N, Yamazaki K, Shimada Y, Tabernero J, Komatsu Y, Sobrero A, Boucher E, Peeters M, Tran B, Lenz H-J, Zaniboni A, Hochster H, Cleary JM, Prenen H, Ohtsu A (2015) Randomized trial of TAS-102 for refractory metastatic colorectal cancer. N Engl J Med 372(20):1909–1919. 10.1056/NEJMoa1414325 25970050 10.1056/NEJMoa1414325

[CR14] Meyerhardt JA, Mayer RJ (2005) Systemic therapy for colorectal cancer. N Engl J Med 352(5):476–487. 10.1056/NEJMra040958 15689586 10.1056/NEJMra040958

[CR15] Moriwaki T, Fukuoka S, Masuishi T, Takashima A, Kumekawa Y, Kajiwara T, Yamazaki K, Esaki T, Makiyama A, Denda T, Hatachi Y, Suto T, Sugimoto N, Enomoto M, Ishikawa T, Kashiwada T, Oki E, Komatsu Y, Tsuji A, Shimada Y (2020) Prognostic scores for evaluating the survival benefit of regorafenib or trifluridine/tipiracil in patients with metastatic colorectal cancer: an exploratory analysis of the REGOTAS study. Int J Clin Oncol 25(4):614–621. 10.1007/s10147-019-01600-0 31838590 10.1007/s10147-019-01600-0

[CR16] Österlund P, Soveri L-M, Isoniemi H, Poussa T, Alanko T, Bono P (2011) Hypertension and overall survival in metastatic colorectal cancer patients treated with bevacizumab-containing chemotherapy. Br J Cancer 104(4):4. 10.1038/bjc.2011.2 21304526 10.1038/bjc.2011.2 PMC3049598

[CR17] Osterlund P, Salminen T, Soveri L-M, Kallio R, Kellokumpu I, Lamminmäki A, Halonen P, Ristamäki R, Lantto E, Uutela A, Osterlund E, Ovissi A, Nordin A, Heervä E, Lehtomäki K, Räsänen J, Murashev M, Aroviita L, Jekunen A (2021) Repeated centralized multidisciplinary team assessment of resectability, clinical behavior, and outcomes in 1086 Finnish metastatic colorectal cancer patients (RAXO): a nationwide prospective intervention study. Lancet Reg Health Eur 3:100049. 10.1016/j.lanepe.2021.100049. (**Members of The RAXO study group are collaborators of this study and are listed in Appendix Table 4B**) 34557799 10.1016/j.lanepe.2021.100049 PMC8454802

[CR18] Pietrantonio F, Miceli R, Rimassa L, Lonardi S, Aprile G, Mennitto A, Marmorino F, Bozzarelli S, Antonuzzo L, Tamburini E, Morano F, Rossini D, Battaglin F, Baretti M, Berenato R, Formica V, Mosconi S, Petrelli F, Ghidini M, Cremolini C (2017) Estimating 12-week death probability in patients with refractory metastatic colorectal cancer: the colon life nomogram. Ann Oncol 28(3):555–561. 10.1093/annonc/mdw627 27864220 10.1093/annonc/mdw627

[CR19] Prahallad A, Sun C, Huang S, Di Nicolantonio F, Salazar R, Zecchin D, Beijersbergen RL, Bardelli A, Bernards R (2012) Unresponsiveness of colon cancer to BRAF(V600E) inhibition through feedback activation of EGFR. Nature 483(7387):100–103. 10.1038/nature10868 22281684 10.1038/nature10868

[CR20] Ranpura V, Pulipati B, Chu D, Zhu X, Wu S (2010) Increased risk of high-grade hypertension with bevacizumab in cancer patients: a meta-analysis. Am J Hypertens 23(5):460–468. 10.1038/ajh.2010.25 20186127 10.1038/ajh.2010.25

[CR21] Scartozzi M, Galizia E, Chiorrini S, Giampieri R, Berardi R, Pierantoni C, Cascinu S (2009) Arterial hypertension correlates with clinical outcome in colorectal cancer patients treated with first-line bevacizumab. Ann Oncol 20(2):227–230. 10.1093/annonc/mdn637 18842611 10.1093/annonc/mdn637

[CR22] Siegel RL, Miller KD, Goding Sauer A, Fedewa SA, Butterly LF, Anderson JC, Cercek A, Smith RA, Jemal A (2020a) Colorectal cancer statistics, 2020. CA A Cancer J Clin 70(3):145–164. 10.3322/caac.21601 10.3322/caac.21601 32133645

[CR23] Siegel RL, Miller KD, Jemal A (2020b) Cancer statistics, 2020. CA A Cancer J Clin 70(1):7–30. 10.3322/caac.21590 10.3322/caac.21590 31912902

[CR24] Tabernero J, Argiles G, Sobrero AF, Borg C, Ohtsu A, Mayer RJ, Vidot L, Moreno Vera SR, Van Cutsem E (2020) Effect of trifluridine/tipiracil in patients treated in RECOURSE by prognostic factors at baseline: An exploratory analysis. ESMO Open 5(4):e000752. 10.1136/esmoopen-2020-000752 32817131 10.1136/esmoopen-2020-000752 PMC7440836

[CR25] Tol J, Nagtegaal ID, Punt CJA (2009) BRAF mutation in metastatic colorectal cancer. N Engl J Med 361(1):98–99. 10.1056/NEJMc0904160 19571295 10.1056/NEJMc0904160

[CR26] Xu J, Kim TW, Shen L, Sriuranpong V, Pan H, Xu R, Guo W, Han S-W, Liu T, Park YS, Shi C, Bai Y, Bi F, Ahn JB, Qin S, Li Q, Wu C, Ma D, Lin D, Li J (2018) Results of a randomized, double-blind, placebo-controlled, phase III trial of Trifluridine/Tipiracil (TAS-102) monotherapy in Asian patients with previously treated metastatic colorectal cancer: the TERRA study. J Clin Oncol 36(4):350–358. 10.1200/JCO.2017.74.3245 29215955 10.1200/JCO.2017.74.3245

[CR27] Yamada Y, Takahari D, Matsumoto H, Baba H, Nakamura M, Yoshida K, Yoshida M, Iwamoto S, Shimada K, Komatsu Y, Sasaki Y, Satoh T, Takahashi K, Mishima H, Muro K, Watanabe M, Sakata Y, Morita S, Shimada Y, Sugihara K (2013) Leucovorin, fluorouracil, and oxaliplatin plus bevacizumab versus S-1 and oxaliplatin plus bevacizumab in patients with metastatic colorectal cancer (SOFT): an open-label, non-inferiority, randomised phase 3 trial. Lancet Oncol 14(13):1278–1286. 10.1016/S1470-2045(13)70490-X 24225157 10.1016/S1470-2045(13)70490-X

[CR28] Yoshino T, Mizunuma N, Yamazaki K, Nishina T, Komatsu Y, Baba H, Tsuji A, Yamaguchi K, Muro K, Sugimoto N, Tsuji Y, Moriwaki T, Esaki T, Hamada C, Tanase T, Ohtsu A (2012) TAS-102 monotherapy for pretreated metastatic colorectal cancer: a double-blind, randomised, placebo-controlled phase 2 trial. Lancet Oncol 13(10):993–1001. 10.1016/S1470-2045(12)70345-5 22951287 10.1016/S1470-2045(12)70345-5

[CR29] Yoshino T, Cleary JM, Van Cutsem E, Mayer RJ, Ohtsu A, Shinozaki E, Falcone A, Yamazaki K, Nishina T, Garcia-Carbonero R, Komatsu Y, Baba H, Argilés G, Tsuji A, Sobrero A, Yamaguchi K, Peeters M, Muro K, Zaniboni A, Lenz H-J (2020) Neutropenia and survival outcomes in metastatic colorectal cancer patients treated with trifluridine/tipiracil in the RECOURSE and J003 trials. Ann Oncol 31(1):88–95. 10.1016/j.annonc.2019.10.005 31912801 10.1016/j.annonc.2019.10.005 PMC7491979

